# Supplementary material for: Cefiderocol Protects against Cytokine- and Endotoxin-Induced Disruption of Vascular Endothelial Cell Integrity in an In Vitro Experimental Model
Source: Antibiotics (Basel). 2022 Apr 26;11(5):581. doi: 10.3390/antibiotics11050581 (PMC9137736; doi:10.3390/antibiotics11050581)

Supplementary Appendix

Cefiderocol protects against cytokine- and endotoxin-induced disruption of vascular endothelial cell integrity in an *in vitro* experimental model.

Supplementary Method

PBMCs were treated with anti CD3/CD28-coated activation beads (Miltenyi Biotech,Bergisch Gladbach, Germany) in a ratio of 1 (cells):10 (beads) -/+ cefiderocol (10mg/L, 40mg/L, 70mg/L) for 3 days. Then, cells were stained with 5µl/ 100.000 cells Annexin-FITC (Thermo Fisher Scientific, Walldorf, Germany) for 15 min at rt and subsequently analyzed on a FACSCanto (Becton Dickinson, Heidelberg, Germany).

Supplementary Figure S1. Cefiderocol does not significantly decrease PBMC viability.

PBMCs were treated with cefiderocol (FDC 10 mg/L, 40 mg/L, 70 mg/L) for 3 daysor left unstimulated. Cells were stained with Annexin-FITC and analyzed on a FACSCanto for percentage of Annexin-positive apoptotic cells. Shown is mean and standard deviation of n=4. Statistical significance was calculated using a one-sided Mann-Whitney U test. There was no statistical significance. Abbreviations: FDC= cefiderocol.

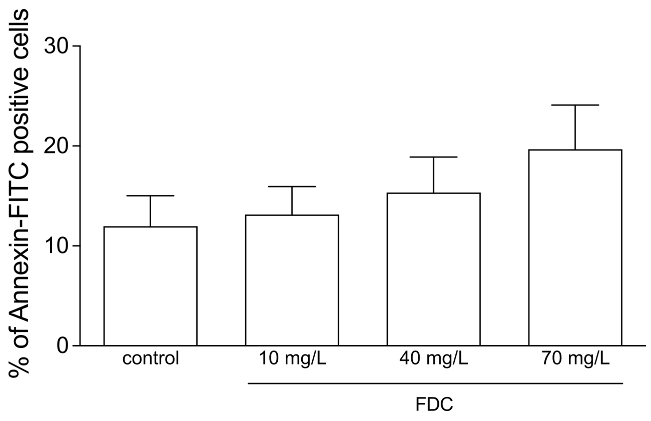

Supplement: Supplementary file 1 [file antibiotics-11-00581-s001.zip › antibiotics-1675130-supplementary.pdf]
